# The Genetic Structures of an Extensively Drug Resistant (XDR) *Klebsiella pneumoniae* and Its Plasmids

**DOI:** 10.3389/fcimb.2018.00446

**Published:** 2019-01-04

**Authors:** Ling Li, Tao Yu, Yanan Ma, Zhongjun Yang, Wenjia Wang, Xiaobo Song, Yu Shen, Tingting Guo, Jian Kong, Mingyu Wang, Hai Xu

**Affiliations:** ^1^State Key Laboratory of Microbial Technology, Microbial Technology Institute, Shandong University, Qingdao, China; ^2^Department of Gastroenterology, Qilu Hospital of Shandong University, Jinan, China; ^3^Department of Stomatology, Qilu Hospital of Shandong University, Qingdao, China; ^4^Department of Medical Biology, Faculty of Health Sciences, University of Tromsø, Tromsø, Norway

**Keywords:** *Klebsiella pneumoniae*, extensively drug resistance, antimicrobial resistance, multidrug resistant plasmid, high throughput sequencing, antimicrobial resistance gene

## Abstract

Multi-, extensively-, and pan-drug resistant bacteria are a threat to our health today, because their wide resistance spectra make their infections difficult to cure. In this work, we isolated an extensively drug resistant (XDR) *Klebsiella pneumoniae* 2-1 strain from the stool sample of a patient diagnosed of colorectal cancer. *K. pneumoniae* 2-1 was found to be resistant to all the antibiotics tested except for cefepime, tigecycline, and ceftazidime-avibactam. By sequencing the complete genome of *K. pneumoniae* 2-1, we found it contains a chromosome of 5.23 Mb and two circular plasmids with the size of 246 and 90 kb. The larger plasmid, pKP21HI1 was found to be a new conjugation-defective plasmid belonging to incompatibility group HI1B and a new sequence type. Further comparative genomics analysis and antimicrobial resistance gene analysis showed that although a great deal of changes took place on the chromosome of *K. pneumoniae* 2-1 in comparison with the reference genome, the extensively drug resistance phenotype of *K. pneumoniae* 2-1 is primarily due to the two multidrug resistant plasmids it contains. This work explains the genetic and mechanistic basis of the extensive drug resistance of *K. pneumoniae* 2-1, and found that plasmids play key roles in the strong antibiotic resistance of bacteria.

## Introduction

*Klebsiella pneumoniae* is the most significant clinical species of the *Klebsiella* genus, and also one of the most frequently observed Gram negative opportunistic pathogens in humans (Podschun and Ullmann, 1998; Navon-Venezia et al., 2017). *K. pneumoniae* can lead to a variety of diseases including urinary tract infections, pneumonia, bacteremia, and liver abscess (Podschun and Ullmann, 1998; Navon-Venezia et al., 2017).

A large catalog of antibiotics such as β-lactams and aminoglycosides are effective in controlling and curing infections caused by *K. pneumoniae* (Bush and Jacoby, 2010; Krause et al., 2016). However, antimicrobial resistance (AMR) caused by the overuse and misuse of antibiotics severely reduces the effectiveness of these antibiotics, leading to increasing difficulties in the treatment of *K. pneumoniae*. For instance, according to the report of a state-wide AMR surveillance program in China, CHINET, the resistance rate of imipenem and meropenem in *K. pneumoniae* increased significantly (from 3.0 to 10.5% for imipenem and from 2.9 to 13.4% for meropenem) between 2005 and 2014 (Hu et al., 2016). Therefore, antibiotic resistance has become a focus in combating *K. pneumoniae* infections in the last decade.

To make the AMR scenario worse, in recent years, infections caused by multidrug-resistant (MDR, non-susceptibility to three or more antimicrobial agent categories), extensively drug-resistant (XDR, susceptible to at most two antimicrobial agent categories), and even pandrug-resistant (PDR, non-susceptibility to all drugs) *K. pneumoniae* have been frequently reported (Falagas et al., 2005, 2008; Leavitt et al., 2010; Karaiskos and Giamarellou, 2014; Huang et al., 2018; Krapp et al., 2018). These *K. pneumoniae* strains are resistant to most or even all the antibiotics used (Magiorakos et al., 2012). The infection of these “superbugs” often leads to immense difficulties in treatment, and sometimes even the death of infected patients (Giske et al., 2008; Hersh et al., 2012). Oftentimes, these strains emerge in ICU wards or on patients treated with organ transplant procedures due to the long-term and extensive use of various antibiotics (Maseda et al., 2014).

Several mechanisms are exploited by bacteria for resistance against antibiotics: mutations on targets of antibiotics may reduce the effectiveness of antibiotics, the alteration and inactivation of antibiotics may be catalyzed by proteins encoded by chromosomal or plasmid-borne genes, mutated porins and efflux pumps may lead to lower levels of antibiotics in the cytoplasm and subsequently reduces their effective dosages (Zhao et al., 2009; Li et al., 2015; Sharma et al., 2016). Antimicrobial resistance genes (ARGs) that take advantage of these resistance mechanisms may be disseminated between bacteria via horizontal gene transfer mechanisms and spread from environmental bacteria to pathogens (Sharma et al., 2016). Therefore, they pose a greater threat to overall human health. To date, a large consortium of ARGs has been discovered that impacts essentially every class of antibiotics. For instance, 51 ARGs have been registered in the Comprehensive Antibiotic Resistance Database (CARD) for carbapenems, one of the last-line antibiotics against Gram-negative bacteria (Nordmann et al., 2011a,b). This list of ARGs include frequently observed *bla*_OXA−48_, *bla*_KPC−2_, *bla*_NDM−1_, *bla*_IMP_, and *bla*_VIM_ in *K. pneumoniae*, and is constantly growing (Kliebe et al., 1985; Bradford et al., 2004; Yu et al., 2012).

The multi-, extensively-, and even pan-drug resistance phenotypes of bacteria is usually the result of horizontal gene transfer that gathers ARGs into one cell (Juhas, 2013). Many mobile genetic elements, including plasmids, integrons, and transposons, are capable of expressing genetically linked and co-expressed ARG arrays that, upon dissemination, bring several ARGs all at once, leading to multidrug resistance (Salabi et al., 2013). In recent years, XDR *K. pneumoniae* turned into an emerging and dangerous pathogen, particularly with the emergence of carbapenem-resistant XDR *K. pneumoniae* (Santino, 2013; Karaiskos and Giamarellou, 2014; Pontikis et al., 2014; Lim et al., 2015). Several therapeutic options are available for XDR *K. pneumoniae*. Fosfomycin has been attempted to treat urinary tract infections and gastrointestinal infections caused by XDR and PDR Enterobacteriaceae (Falagas et al., 2010; Leavitt et al., 2010; Braun et al., 2018). Colistin and tigecycline are the last resort treatments for serious carbapenem-resistant *K. pneumoniae* infections (Olaitan et al., 2014; Piedra-Carrasco et al., 2018). However, AMR against these antibiotics arose, such as *fosA3* for fosfomycin, *mcr-1* for polymyxin, and *tetA* mutation for tigecycline (Jiang et al., 2015; Liu et al., 2015; Du et al., 2018). Further successful attempts for the treatment of XDR and PDR *K. pneumoniae* were made, including antibiotic combination therapies such as using double carbapenems (Piedra-Carrasco et al., 2018), tigecycline plus meropenem (Lim et al., 2015), and β-lactam/β-lactamase inhibitor combos such as ceftazidime plus avibatam (Schimmenti et al., 2018).

Plasmids are the most important carriers for ARGs in MDR *K. pneumoniae*, and are found in almost all antimicrobial resistant *K. pneumoniae* isolates (Dolejska et al., 2013; Freire Martín et al., 2014; Pitout et al., 2015; Navon-Venezia et al., 2017). A total of 306 complete *K. pneumoniae* plasmid sequences are currently available at Genbank (Navon-Venezia et al., 2017). In particular, pKpQIL and pKPN3 that belong to incompatibility group FIIK are commonly found in *K. pneumoniae* belonging to the epidemic clonal group CG258 (Chen et al., 2014; Navon-Venezia et al., 2017). These two plasmids contain important ARGs such as *bla*_KPC−2_ and *bla*_KPC−3_, and are frequently reported globally (Leavitt et al., 2010; García-Fernández et al., 2012; Chen et al., 2013, 2014).

In this study, *K. pneumoniae* 2-1, an XDR *K. pneumoniae*, was isolated from the stool sample of a patient with colorectal cancer. Two large plasmids were found in this strain. The full genomic sequence of this strain, including both chromosomal DNA sequence and the DNA sequence of two plasmids it harbors, were obtained. Comparative genomic sequence analysis showed one of the plasmids, pKP21HI1, is a new plasmid belonging to IncHI1B and forms a new sequence type. Further analysis of both chromosomal and plasmid-borne ARGs showed the XDR phenotype in this strain is primarily contributed by the ARGs harbored by the two multidrug resistance plasmids. Findings of this work suggest that attention should be focused on multidrug resistance plasmids, as they could determine the XDR phenotype of *K. pneumoniae*, are highly mobile, and are difficult to contain.

## Materials and Methods

### Bacterial Strain

*K. pneumoniae* 2-1 used in this work was isolated from the stool sample of a colorectal cancer patient in Qilu Hospital, Jinan. The patient was treated for lasting abdominal pain and constipation, and was initially prescribed with levofloxacin because he was misdiagnosed of enteritis. Strain identification was performed by analyzing the 16S rDNA sequence of this strain. *K. pneumoniae* HS11286 strain is a previously identified model clinical strain that was generously gifted from Prof. Hongyu Ou from Shanghai Jiao Tong University (Bi et al., 2015).

### Antibiotic Susceptibility Testing

All antibiotic susceptibility assays were performed following CLSI standard (Wayne, 2018b). Disk diffusion assays were performed as previously documented (Vading et al., 2011). Minimum Inhibition Concentrations (MICs) was determined with the agar dilution method as previously reported (Wiegand et al., 2008). *Escherichia coli* ATCC 25922 was used as a reference strain as recommended by CLSI standard (Wayne, 2018a).

### Transfer of Plasmids to *E. coli* DH5α

To transfer plasmids of *K. pneumoniae* 2-1 to *E. coli* DH5α, plasmids were extracted from *K. pneumoniae* 2-1, and transferred to the recipient *E. coli* DH5α strain by either chemical transformation or electrotransformation. Successful transfer was determined by trimethoprim resistance (for pKP21HI1) or tetracycline resistance (for pKP21AC2). Further validation was performed by extracting plasmids from plasmid-containing *E. coli* DH5α and subsequent PCR amplification of *aadA2, sul1, dfrA12* for pKP21HI1 or *sul2, floR* for pKP21AC2.

### Conjugation Assays

Conjugations assays were performed in order to test whether plasmids in *K. pneumoniae* 2-1 can be transmitted via conjugal transfer, following previously published procedures (Borgia et al., 2012). *E. coli* J53 was used as the recipient strain. Successful conjugal transfer of plasmid was determined by the dual resistance of sodium azide and trimethoprim/streptomycin/tetracycline/chloramphenicol.

### Whole Genome Sequencing

The total DNA of *K. pneumoniae* 2-1 was extracted using the SDS method as previously reported (Natarajan et al., 2016). Total DNA samples were shredded into 10 kb fragments using g-TUBE (Covaris Inc., Woburn, MA, US) to construct a 10 kb SMRT Bell library for PacBio sequencing. Total DNA samples were shredded with ultrasonication to construct a 350 bp library for Illumina sequencing. Libraries were, respectively, sequenced with a PacBio Sequel system (Pacific Biosciences of California, Inc., Menlo Park, CA, US) and an Illumina HiSeq X 10 system (Illumina Inc., San Diego, CA, US) at PE150 mode. DNA sequences were assembled with SMRT Link v5.1.0 software (Ardui et al., 2018). Gaps were manually closed by PCR amplifying gap-containing DNA using primers targeting each end of the gap and subsequent sequencing. Sequence data were deposited in Genbank, with accession numbers of CP031562, CP031563, and CP031564.

### Bioinformatics

Gene model prediction was performed using GeneMarkS Version 4.1.7 (Besemer et al., 2001). The predicted gene models were subsequently annotated with Gene Ontology (GO) (Blake et al., 2015), Kyoto Encyclopedia of Genes and Genomes (KEGG) (Kanehisa and Goto, 2000), Cluster of Orthologous Groups of proteins (COG) (Natale et al., 2000), Non-Redundant Protein Database (NR) (Li et al., 2002), Pfam (Punta et al., 2011), and Swiss-Prot (Boeckmann et al., 2003) databases.

For ARG analysis, the Resistance Gene Identifier (RGI) v4.1.0 tool of The CARD was used (Jia et al., 2017).

For classification of *K. pneumoniae* 2-1, the multilocus sequencing typing method was used as previously reported (Diancourt et al., 2005).

For classification of plasmids, the PlasmidFinder and pMLST webtools were used (Carattoli et al., 2014).

Phylogenetic analysis was performed using the maximum likelihood (ML) method with the MEGA 7.0.21 software (Hall, 2013).

Comparative genomics analysis was performed using the MUMmer v3.2.3 software (Kurtz et al., 2004). InDels were identified using the LASTZ v1.03.54 software.

### Ethics

This study was carried out in accordance with the recommendations of Scientific Ethics Committee of Qilu Hospital of Shandong University. The protocol was approved by the Scientific Ethics Committee of Qilu Hospital of Shandong University. All subjects gave written informed consent in accordance with the Declaration of Helsinki.

## Results

### Isolation of an XDR *K. pneumoniae*

A *K. pneumoniae* 2-1 strain was isolated from the stool sample of a 36-year-old male patient with colorectal cancer. Antimicrobial susceptibility testing of 23 antibiotics was performed using both K-B disk diffusion assay and agar dilution method, showing it is an XDR strain that is resistant to nearly all antibiotics tested (Table 1). A more conservative approach was undertaken to determine the susceptibility of *K. pneumoniae* 2-1: if the strain is sensitive to an antibiotic using either K-B disk diffusion assay or agar dilution method, the strain is considered sensitive to this antibiotic. The antibiotics that *K. pneumoniae* 2-1 is non-susceptible to include β-lactams (ampicillin, amoxicillin, ceftazidime, cefotaxime, cefoxitin, cefoperazone), carbapenem (imipenem), aminoglycosides (spectinomycin, kanamycin, streptomycin), quinolones (gatifloxacin, ciprofloxacin, nalidixic acid), diaminopyrimidine (trimethoprim), sulfonamide (sulfisoxazole), rifampicin (rifampicin), macrolide (erythromycin), phenicol (chloramphenicol), tetracycline (tetracycline), and polymyxins (polymyxin B and polymyxin E). *K. pneumoniae* 2-1 was found to be susceptible to only two antibiotics tested: a fourth generation cephalosporin cefepime and a last-line antibiotic tigecycline. Out of the three β-lactam/β-lactamase inhibitor combos, *K. pneumoniae* 2-1 was found to be sensitive to only one combo: ceftazidime-avibactam. The antibiotic susceptibility of the reference clinical *K. pneumoniae* HS11286 strain was also performed as a reference (Table 1).

**Table 1:** The antibiotic susceptibility of *K. pneumoniae* 2-1 and *K. pneumoniae* HS11286.

**Antibiotic class**	**Antibiotics**	***K. pneumoniae*** **2–1**		***K. pneumoniae*** **HS11286**	
		**Inhibition zone (mm)**	**MIC (μg/ml)**	**Inhibition zone (mm)**	**MIC (μg/ml)**
β-lactam	Ampicillin (AMP)	R(0)	R(>512)	R(0)	R(>512)
	Amoxicillin-Clavulanate (AMO)	R(0)	R(64/32)	R(0)	R(256/128)
	Ceftazidime-Avibactam	R(19)	S(0.5/4)	S(22)	S(2/4)
	Piperacillin-Tazobactam	R(15)	R(256/4)	R(0)	R(>256/4)
	Cefepime (FEP)	SDD(24)	S(0.5)	R(12)	R(32)
	Ceftazidime (CAZ)	R(9)	R(32)	R(0)	R(256)
	Cefotaxime (CTX)	R(20)	R(32)	R(0)	R(256)
	Cefoxitin (CFX)	R(0)	R(512)	R(0)	R(512)
	Cefoperazone (CFP)	I(18)	I(32)	R(0)	R(>512)
Carbapenem	Imipenem (IPM)	R(9)	I(2)	R(0)	R(16)
Aminoglycoside	Spectinomycin (SPE)	I(16)	–	R(14)	–
	Kanamycin (KAN)	R(0)	R(>512)	R(0)	R(>512)
	Streptomycin (STR)	R(8)	–	R(0)	–
Quinolone	Gatifloxacin (GAT)	R(0)	R(32)	I(15)	R(>512)
	Ciprofloxacin (CIP)	R(9)	R(128)	I(16)	S(1)
	Nalidixic acid (NAL)	R(0)	R(>512)	R(0)	R(>512)
Diaminopyrimidine	Trimethoprim (TMP)	R(0)	R(>512)	R(0)	R(>512)
Sulfonamide	Sulfisoxazole (SFI)	R(0)	R(>512)	R(0)	R(>512)
Rifampicin	Rifampicin (RFP)	R(0)	–	R(11)	–
Macrolide	Erythromycin (EM)	R(0)	–	R(0)	–
Phenicol	Chloramphenicol (CHL)	R(0)	R(256)	R(12)	R(128)
Tetracycline	Tetracycline (TET)	R(0)	R(64)	R(10)	R(512)
Glycylcycline	Tigecycline (TIG)	S(20)	S(1)	I(17)	S(1)
Polymyxin	Polymyxin B (PMB)	N	R(4)	N	S(2)
	Polymyxin E (PME)	N	R(4)	N	S(2)

*–, MIC breakpoint unavailable in CLSI standard*.

*N, Disk diffusion test not recommended in CLSI standard*.

### The Genetic Features of *K. pneumoniae* 2-1

**Figure 1:**
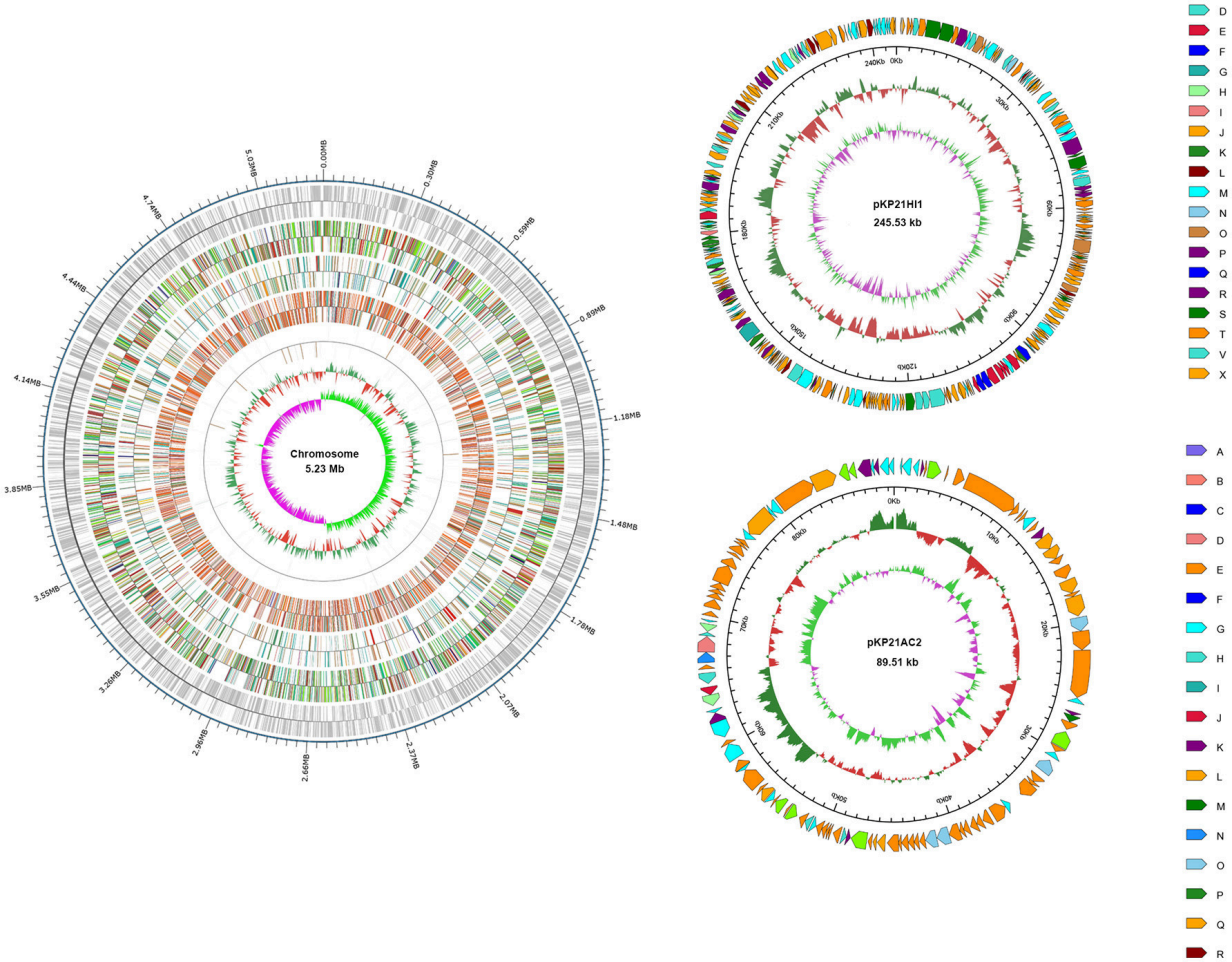
Genome map of *K. pneumoniae* 2-1 chromosome and plasmids. For chromosome, from outside to inside, each ring represents encoding genes, COG, KEGG, GO, ncRNA, GC content, and genomic GC skew value. For plasmids, from outside to inside, each ring represents COG, GC content, and genomic GC skew value. Different color represents different annotation.

Total DNA was extracted from *K. pneumoniae* 2-1 and sequenced on a PacBio system. A circular genomic DNA with the size of 5,230,042 basepairs was assembled. In addition, two circular plasmids, respectively, with the size of 245,534 and 89,508 basepairs were found. The plasmids were subsequently denominated pKP21HI1 and pKP21AC2. The GC content for the genomic DNA, pKP21HI1, and pKP21AC2 are, respectively, 57.57, 51.43 and, 52.92%. The numbers of genes encoded by the genomic DNA, pKP21HI1 and pKP21AC2 are respectively 4972, 268, and 114. Functional annotation was performed with a variety of databases including GO, KEGG, COG, NR, Pfam and Swiss-Prot (Figure 1, Table S1).

### Classification of *K. pneumoniae* 2-1 and its Plasmids

MLST classification of *K. pneumoniae* 2-1 was performed using the PubMLST database based on genetic variation in seven housekeeping genes (*rpoB, gapA, mdh, pgi, phoE, infB*, and *tonB*) (Diancourt et al., 2005), confirming a 2-9-2-1-13-1-4 configuration and supporting the subsequent classification of *K. pneumoniae* 2-1 as ST726.

**Figure 2:**
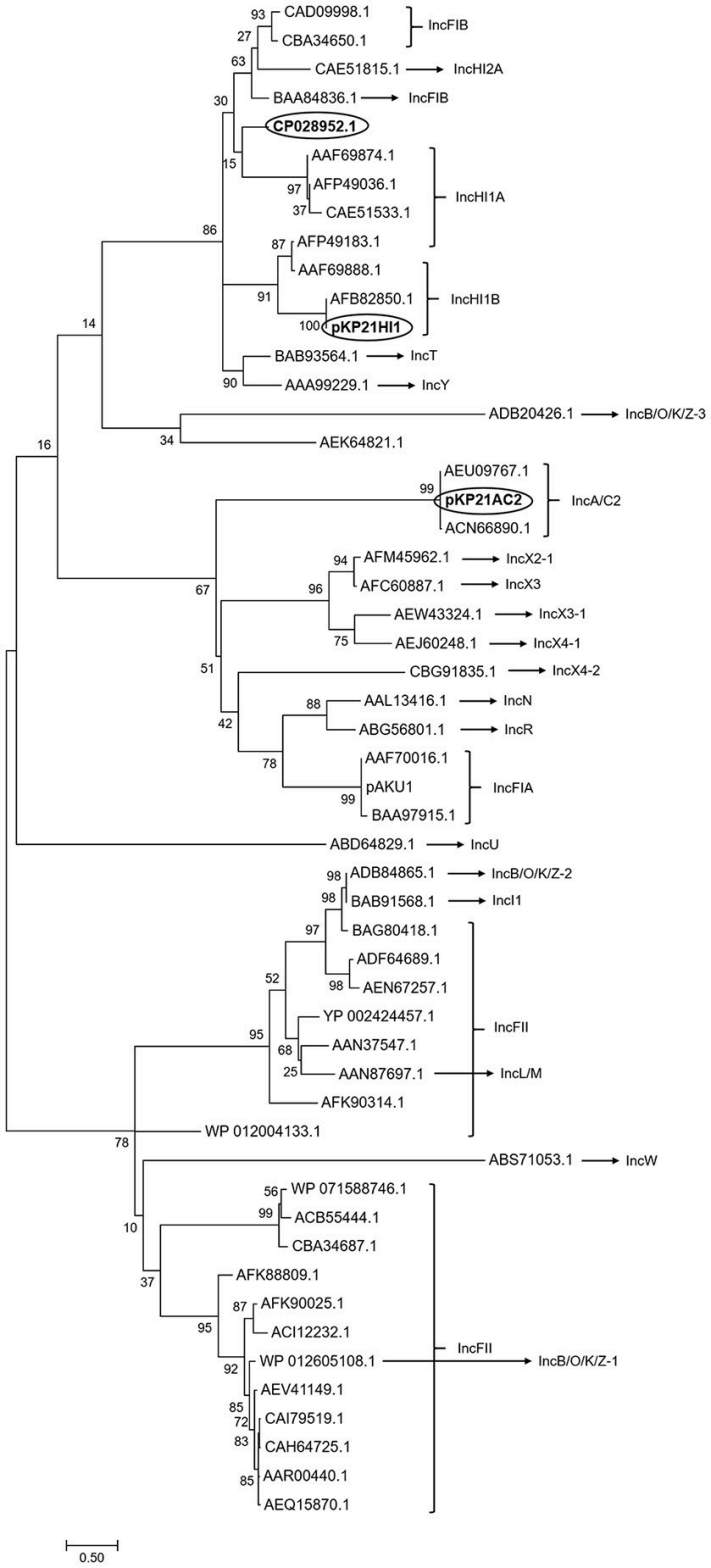
Phylogenetic analysis of pKP21AC2 and pKP21HI1. Phylogenetic tree of replicon sequence of pKP21HI1, pKP21AC2, and plasmids belonging to different incompatible group was shown. The phylogenetic tree was calculated from 500 individual trees. Bootstrap values were shown on each node. Bar: evolutionary distance of 0.5. Circled are the two plasmids found in *K. pneumoniae* 2-1 and the most similar plasmid to pKP21HI1.

The classification of pKP21HI1 and pKP21AC2 was performed based on the typing of its replicon sequences, using the PlasmidFinder database (https://cge.cbs.dtu.dk/services/PlasmidFinder/). These two plasmids were, respectively, classified as IncHI1B and IncA/C2 plasmids, suggested by the presence of *repH1B* and *repA*. This classification is further confirmed by phylogenetic analysis of replicon sequences of pKP21HI1 and pKP21AC2, together with replicon sequences of known plasmids of different incompatible groups (Figure 2). This phylogenetic analysis suggested that pKP21HI1 forms a clade with other IncHI1B plasmids, while pKP21AC2 forms a clade with other IncA/C2 plasmids. The sequence types of these two plasmids were further determined with the plasmid multilocus sequence typing (pMLST) analysis with the pMLST webtool (Carattoli et al., 2014). The pKP21AC2 plasmid was confirmed to be a ST-3 plasmid belonging to the IncA/C incompatibility group based on a *repA*-*parA*-*parB*-*A053* configuration of 2-2-2-1. However, only one (*hcm1043*) of the six (*hcm1043, hcm1099, hcm1064, hcm1116, hcm1178ac, hcm1259*) genes used for sequence typing of IncHI1 plasmids was found in pKP21HI1, suggesting it belongs to a new sequence type of IncHI1 plasmids. Indeed, pMLST analysis was unable to assign pKP21HI1 to any sequence type, and BLAST search of pKP21HI1 in Genbank showed the most similar plasmid (*Klebsiella aerogenes* strain AR_0161 plasmid unnamed, Genbank accession number CP028952.1) is 206 kbp larger than pKP21HI1.

### Comparative Genomic Analysis of *K. pneumoniae* 2-1 and the Reference *K. pneumoniae* Strain HS11286

A comparison between *K. pneumoniae* 2-1 and the reference clinical *K. pneumoniae* HS11286 chromosomal DNA found significant differences between the two genomes. A total of 22,360 SNPs were found between the two genomes, including 19,659 SNPs that fall into the CDS region, of which 3,789 are non-synonymous. In particular, 25 SNPs lead to premature termination of the gene products (Table S2). A total of 116 InDels were found in *K. pneumoniae* 2-1 in comparison with *K. pneumoniae* HS11286, leading to the frameshift of 102 gene products and the premature termination of 1 CDS (Table S2). One hundred and sixteen structural variations were found in *K. pneumoniae* 2-1, including 34 complex InDels, 34 deletions, 36 insertions, and 1 inversion (Table S2). These changes do not appear to affect the antimicrobial resistance of *K. pneumoniae* 2-1.

### pKP21HI1 as a new Conjugation-Defective Multidrug Resistant Plasmid

The analysis of the presence of ARGs in both pKP21HI1 and its most similar plasmid CP028952.1 showed the presence of, respectively, 18 and 13 ARGs (Table 2), suggesting both plasmids are multidrug resistant plasmids. Comparison between these two plasmids showed significant differences. In comparison with CP028952.1, a large fragment (206 kbp) is missing in pKP21HI1. pKP21HI1 codes for 50 genes that are absent in CP028952.1, while CP028952.1 codes for 278 genes that are absent in pKP21HI1 (Table S3). Of particular interest, the genes absent in pKP21HI1 include 29 transposase/intergrase-coding genes and 13 conjugation/ plasmid transfer protein-coding genes (*traN, traX, traG, traF, traI, traD, traE, traB, traV*). This is a strong suggestion that CP028952.1 is a more mobile plasmid than pKP21HI1. Indeed, no conjugal protein-coding genes could be found in pKP21HI1, and conjugation assays in attempt to transfer pKP21HI1 to *E. coli* J53 were unsuccessful. These results suggest that pKP21HI1 is a conjugation-defective multidrug resistant plasmid that limits its mobility between bacteria.

**Table 2:** Comparison of ARGs in pKP21HI1 and CP028952.1.

**Resistant antibiotics**	**pKP21HI1**	**CP028952.1**
Aminoglycosides	*aph-(6′)-Ia*	*aac-(6′)-IId*
	*armA*	*aph-(6′)-Id*
	*aadA2*	*aph-(3′)-Ib*
	*aac-(3′)-IIa*	*aac-(3′)-IIa*
Quinolones	*qnrB4*	*aac-(6′)-Ib-cr*
	*aac-(6′)-Ib-cr*	
β-lactam	*bla*_OXA-1_	*bla*_OXA-1_
	*bla*_TEM-1_	*bla*_TEM-1_
	*bla*_DHA-1_	*bla*_IMP-4_
Sulfonamides	*sul1*	*sul1*
Phenicols	*catB3*	*catB3*
Macrolides	*mphD*	
	*msrE*	
Rifamycin	*arr-3*	*arr-3*
Diaminopyrimidines	*dfrA12*	
Efflux pump	*vgaC*	*vgaC*

### ARGs Harbored by *K. pneumoniae* 2-1 and Its Plasmids

*K. pneumoniae* 2-1 and its plasmids are annotated for ARGs with the CARD database (Table 3). A total of 33 ARGs were found, of which 23 are plasmid-borne while the rest are chromosomal. By comparing the ARG catalog of *K. pneumoniae* 2-1 and *K. pneumoniae* HS11286, we found little difference except that *K. pneumoniae* 2-1 encodes two additional quinolone resistance genes *oqxA* and *oqxB*. The mutations of several genes that lead to antibiotic resistance were found in the chromosome of *K. pneumoniae* 2-1, including mutation on elongation factor Tu that leads to resistance to pulvomycin, mutation in *uhpT* that confers resistance to fosfomycin, *parC* and *gyrA* mutation that leads to resistance to quinolones, *marR* mutation that leads to multidrug resistance, and mutation in *ftsI* that confers resistance to β-lactams. Therefore, *K. pneumoniae* 2-1 chromosome encodes genes responsible for quinolone and β-lactam resistance phenotypes found in this work (Table 1). pKP21AC2 hosts 5 ARGs while pKP21HI1 hosts 18 ARGs, including genes for aminoglycoside resistance, quinolone resistance, β-lactam resistance, sulfonamide resistance, chloramphenicol resistance, tetracycline resistance, macrolide resistance, rifamycin resistance, and trimethoprim resistance. The genes encoded by these two plasmids were found responsible for aminoglycoside, sulfonamide, chloramphenicol, tetracycline, macrolide, rifamycin, and trimethoprim resistance that were found in phenotypic analysis but were not encoded by the chromosome. Two class I integrons were found in pKP21HI1, each containing an ARG-rich gene cassette array in the organization of *dfrA12-aadA2* and *aac-(6')-Ib-cr-bla*_OXA−1_-*catB3-arr-3* (Figure S1). These findings suggest that the XDR phenotype is a result of the presence of the two multidrug resistance plasmids harbored by *K. pneumoniae* 2-1.

**Table 3:** ARGs in *K. pneumoniae* 2-1 and *K. pneumoniae* HS11286.

**Resistant antibiotics**	***K. pneumoniae* HS11286 chromosome**	***K. pneumoniae* 2-1 chromosome**	**pKP21HI1**	**pKP21AC2**
Aminoglycosides			*aph-(6′)-Ia*	*aph-(3′)-Ib*
			*armA*	*aph-(6′)-Id*
			*aadA2*	
			*aac-(3′)-IIa*	
Quinolones	*patA*	*oqxA*	*qnrB4*	
		*oqxB*	*aac-(6′)-Ib-cr*	
		*patA*		
β-lactam	*bla*_SHV−11_	*bla*_SHV−11_	*bla*_TEM−1_	
			*bla*_DHA−1_	
			*bla*_OXA−1_	
Sulfonamides			*sul1*	*sul2*
Phenicols			*catB3*	*floR*
Tetracycline				*tetC*
Macrolides			*mphD*	
			*msrE*	
Rifamycin			*arr-3*	
Diaminopyrimidines			*dfrA12*	
Fosfomycin	*fosA6*	*fosA6*		
Nitromidazole	*msbA*	*msbA*		
Efflux pump	*adeF*	*adeF*	*vgaC*	
	*emrB*	*emrB*		
	*acrA*	*acrA*		

To further confirm the roles of the plasmids in antimicrobial resistance, we extracted the plasmids and transferred them to *E. coli* DH5α. The antibiotic susceptibility tests were performed, showing that *E. coli* DH5α containing pKP21HI1 is resistant to β-lactams, aminoglycosides, rifamycin, macrolides, quinolones, trimethoprim, and sulfonamides, while *E. coli* DH5α containing pKP21AC2 is resistant to aminoglycosides, quinolones, rifamycin, macrolides, sulfonamides and tetracycline.

## Discussion

XDR *K. pneumoniae* is an eminent threat to human health in an era when the discovery of new antibiotics lags behind the emergence and dissemination of antimicrobial resistance. Therefore, understanding the genetic and mechanistic basis for XDR becomes crucial, which may provide a hint on finding solutions to prevent its spread. In this work, with high throughput sequencing, we obtained the complete sequence of an XDR *K. pneumoniae* 2-1 strain isolated from a patient with colorectal cancer, and identified the genetic basis for its XDR phenotype.

*K. pneumoniae* 2-1 was found resistant to nearly all antibiotics tested except for cefepime, tigecycline, and ceftazidime-avibactam. A total of 33 ARGs and 6 gene mutations responsible for AMR were found on the chromosome and plasmids of *K. pneumoniae* 2-1. ARGs for all the resistant antibiotics were found except for carbapenems and polymyxins. The presence of *bla*_OXA−1_ would have been responsible for carbapenem resistance, as *bla*_OXA−1_-containing strains that are resistant to carbapenems have been previously reported (Sugumar et al., 2014). However, the *bla*_OXA−1_-harboring pKP21HI1 didn't increase the resistance of *E. coli* DH5α to imipenem (Table 4), making this hypothesis unlikely true. Considering neither plasmid was able to increase the resistance of *E. coli* DH5α (Table 4), it is likely that a yet unknown chromosomal mechanism is responsible for the carbapenem resistance of *K. pneumoniae* 2-1. *K. pneumoniae* 2-1 also encodes a series of pmr phosphoethanolamine transferases (*basR, arnA, eptA, ugd, pmrF*) that involve in polymyxin resistance, when we set the search parameter for CARD database as “Loose”. Therefore, all the resistance phenotypes found in this work are accounted for except for carbapenems, all of which involve one or several ARGs.

**Table 4:** The antibiotic susceptibility of *E. coli* DH5α (DH5α), *E. coli* DH5α containing pKP21HI1 (DH5α-pHI1) and *E. coli* DH5α containing pKP21AC2 (DH5α-pAC2).

**Antibiotic class**	**Antibiotics**	**Inhibition zone (mm)**			**MIC (μg/ml)**		
		**DH5α**	**DH5α-pHI1**	**DH5α-pAC2**	**DH5α**	**DH5α-pHI1**	**DH5α-pAC2**
β-lactam	Ampicillin	S(27)	R(0)	S(25)	S(1)	R(256)	S(1)
	Amoxicillin-Clavulanate	S(29)	R(7)	S(20)	S(8/4)	R(64/32)	S(4/2)
	Ceftazidime-Avibactam	S(34)	S(36)	S(35)	S(0)	S(0)	S(0)
	Piperacillin-Tazobactam	S(26)	S(24)	I(19)	S(8/4)	S(8/4)	S(4/4)
	Cefepime	S(36)	S(32)	S(32)	S(0)	S(0)	S(0)
	Ceftazidime	S(34)	S(26)	S(23)	S(0)	S(0.25)	S(0)
	Cefotaxime	S(37)	S(29)	S(29)	S(0)	S(0.125)	S(0)
	Cefoxitin	S(22)	R(7)	S(20)	I(16)	R(64)	I(16)
	Cefoperazone	S(35)	S(30)	S(28)	S(0)	S(0.5)	S(0)
Carbapenem	Imipenem	S(25)	S(26)	S(35)	S(0)	S(0.25)	S(0.25)
Aminoglycoside	Spectinomycin	S(29)	S(22)	S(30)	–	–	–
	Kanamycin	S(30)	R(0)	S(26)	S(0.25)	R(128)	S(32)
	Streptomycin	S(26)	S(26)	R(10)	–	–	–
Quinolone	Gatifloxacin	S(30)	S(29)	S(35)	S(0)	S(0.125)	S(0.125)
	Ciprofloxacin	S(31)	S(30)	S(25)	S(0)	S(0.125)	S(0.125)
	Nalidixic acid	I(14)	R(0)	R(13)	R(128)	R(256)	R(256)
Diaminopyrimidine	Trimethoprim	S(35)	R(0)	S(35)	S(4)	R(256)	R(128)
Sulfonamide	Sulfisoxazole	S(23)	S(40)	R(0)	R(512)	R(512)	R(512)
Rifampicin	Rifampicin	S(23)	R(12)	I(18)	–	–	–
Macrolide	Erythromycin	S(18)	R(9)	R(0)	–	–	–
Phenicol	Chloramphenicol	S(32)	S(28)	R(11)	S(2)	S(2)	S(8)
Tetracycline	Tetracycline	S(27)	S(26)	I(12)	S(2)	S(2)	R(64)
Glycylcycline	Tigecycline	S(20)	S(30)	S(24)	S(0)	S(0.25)	S(0.25)
Polymyxin	Polymyxin E	N	N	N	S(1)	S(1)	S(1)

*–, MIC breakpoint unavailable in CLSI standard*.

*N, Disk diffusion test not recommended in CLSI standard*.

One particularly interesting finding in this work is that the majority of ARGs are encoded by the two plasmids of *K. pneumoniae* 2-1. The resistance to aminoglycosides, sulfonamides, chloramphenicol, tetracycline, macrolides, rifamycin, and trimethoprim are not encoded by the chromosome, and completely depend on the presence of the two plasmids. Therefore, the two multidrug plasmids play a key role in the XDR phenotype of *K. pneumoniae* 2-1, which is worrisome as plasmids can easily transfer between bacteria, disseminating ARGs.

The analysis of *E. coli* DH5α strains, respectively, containing pKP21HI1 and pKP21AC2 further confirmed the role of the two plasmids in antimicrobial resistance. pKP21HI1 but not pKP21AC2 contains ARGs for β-lactams and diaminopyrimidines, while the pKP21HI1-containing *E. coli* DH5α but not the pKP21AC2-containing *E. coli* DH5α is resistant to β-lactams and trimethoprim. pKP21AC2 but not pKP21HI1 contains ARGs for tetracycline, while the pKP21AC2-containing *E. coli* DH5α but not the pKP21HI1-containing *E. coli* DH5α is resistant to tetracycline. Both plasmids contain ARGs for aminoglycosides and sulfonamides, while both the pKP21HI1-containing *E. coli* DH5α and the pKP21AC2-containing *E. coli* DH5α are resistant to these classes of antibiotics. Neither plasmids contain ARGs for carbapenems, while neither plasmid-containing *E. coli* DH5α strain is resistant to carbapenems, further agreeing with the hypothesis that a chromosomal mechanism could be responsible for carbapenem resistance. Although both plasmids carry ARGs for chloramphenicol, neither plasmid-containing *E. coli* DH5α strain was shown to be resistant to chloramphenicol. Therefore, the resistance to chloramphenicol requires the presence of both plasmids in the cell. The disagreement between the presence of ARGs in the plasmids and the antimicrobial resistance phenotype is that although pKP21AC2 lacks ARGs for quinolones, macrolides and rifamycin, pKP21AC2-harboring *E. coli* DH5α is resistant to nalidixic acid, erythromycin, and rifamycin. The resistance of pKP21AC2-harboring *E. coli* DH5α to nalidixic acid is due to the innate resistance of *E. coli* DH5α to this antibiotic (Table 4). By further investigations of pKP21AC2 sequences, we found that pKP21AC2 contains a gene that may encode a SoxR mutant conferring resistance to rifamycin (sequence identity 39.71%) and a gene that may encode a mutated repressor (NalD) that could lead to the overexpression of the MexAB-OprM efflux pump for resistance to macrolides (sequence identity 36.17%). Therefore, we hypothesize that these distant homologs to existing antimicrobial resistance determinants may be responsible for rifamycin and macrolide resistance in pKP21AC2. It needs to be addressed that in general, plasmid-harboring *E. coli* DH5α showed lower levels of antimicrobial resistance in comparison with *K. pneumoniae* 2-1. This phenomenon suggests that the combination of MDR plasmids and chromosomal mutations can significant increase the overall antibiotic resistance level.

The findings in this work showed the importance of plasmids in XDR. Out of the two multidrug resistance plasmids identified in *K. pneumoniae* 2-1, pKP21HI1 is a new plasmid that differs significantly to all previously known plasmids. Indeed, the most similar plasmid CP028952.1 from *Klebsiella aerogenes* is 206 kb larger than pKP21HI1, and analysis of its genetic content revealed that it belongs to a new sequence type of incompatibility group HI1B. This plasmid encodes 18 ARGs that leads to resistance of 9 major classes of antibiotics, as well as two intact Class 1 integrons, suggesting its significant role in AMR and its dissemination. Fortunately, this plasmid appears to be conjugation-defective, therefore limiting its mobility. Nevertheless, it can still disseminate between bacteria via other processes such as natural transformation.

The identification of the genetic and mechanistic basis of XDR in *K. pneumoniae* 2-1 stresses the role of multidrug resistance plasmids in XDR. Comparison of *K. pneumoniae* 2-1 and the reference genome of *K. pneumoniae* HS11286 showed only minor differences in antimicrobial resistance on the chromosomal level. Therefore, although the possibility of chromosomal MDR *K. pneumoniae* is present and reported (Huang et al., 2017; Mathers et al., 2017), proposal can be made that the external antibiotic pressure favors concentrating ARGs to existing strains in the form of multidrug plasmids, rather than leading to the evolvement of strains with chromosome-encoded XDR. With this proposal, further suggestion can be made that cutting off transmission of plasmids may be a good approach in delaying the appearance of XDR and PDR strains.

## Conclusions

In this work, with the analysis of the genetic features of an XDR *K. pneumoniae* 2-1 strain isolated from the stool sample of a patient diagnosed of colorectal cancer, we found and identified a new conjugation-defective multidrug resistant pKP21HI1 plasmid that belongs to incompatibility group HI1B and a new sequence type. With further analysis of the antimicrobial resistance phenotype of *K. pneumoniae* 2-1 and the distribution of ARGs on its chromosome and two plasmids, conclusions can be made that the XDR phenotype should be contributed to primarily its plasmids rather than chromosome. Research from this work stressed the importance of multidrug resistant plasmids in leading to extensively- and pan-drug resistance, and lead to the proposal that efforts should be made to control plasmid dissemination in order to delay the appearance of the XDR or PDR pathogens.

## Author Contributions

LL, YM, and WW performed experiments. TY performed strain isolation. LL, ZY, XS, YS, TG, JK, MW, and HX performed bioinformatical analysis. LL, MW, and HX wrote the manuscript. MW and HX conceived of the study. HX oversaw the project. All authors read and approved the manuscript.

### Conflict of Interest Statement

The authors declare that the research was conducted in the absence of any commercial or financial relationships that could be construed as a potential conflict of interest.
