# Supplementary material for: The Genetic Structures of an Extensively Drug Resistant (XDR) Klebsiella pneumoniae and Its Plasmids
Source: Front Cell Infect Microbiol. 2019 Jan 4;8:446. doi: 10.3389/fcimb.2018.00446 (PMC6328971; doi:10.3389/fcimb.2018.00446)
Supplement: Supplementary file 2 [file Table_2.doc]

**Table S2. Comparison of *K. pneumoniae* 2-1 genomic features with *K. pneumoniae* HS11286**.

| **Type of genomic change** | **Position** | **Affected genes** |
| --- | --- | --- |
| Premature termination caused by SNP | 118346  452069  511004  574056  677483  1152349  1437600  1476442  1660199,1660215,1660219,1660239  1741378  2019393  2632635  3265346  3300450  3543910  3677571  4396599  4958674  5198730  5258121  5281599  5329926 | Vitamin B12/cobalamin outer membrane transporter  DNA mismatch repair protein MutL  hypothetical protein  Sugar ABC transporter  hypothetical protein  hypothetical protein  hypothetical protein  hypothetical protein  hypothetical protein  Anaerobic C4-dicarboxylate transporter  Ribosomal-protein-alanine acetyltransferase  hypothetical protein  Putative ribosomal large subunit pseudouridine synthase A  hypothetical protein  Bifunctional phosphoribosyl-AMP cyclohydrolase/phosphoribosyl-ATP pyrophosphatase protein  Ferric iron-catecholate outer membrane transporter  Outer membrane protein for export and assembly of type 1 fimbriae  hypothetical protein  hypothetical protein  RelE family toxin-antitoxin system  DNA-damage-inducible protein D  ATP synthase F0, A subunit |
| InDel (Insertion) | 2612876-2612877  2002594-2002596  928758-928759  2420907-2420908  950475-950476  3037914-3037915  3308219-3308220  2282045-2282046  2434750-2434751  3457632-3457633  3511791-3511792  227478-227479  2197505-2197506  3495762-3495763 | hypothetical protein  hypothetical protein  low-affinity _L_-arabinose transport system proton symport component  hypothetical protein  hypothetical protein  hypothetical protein  putative tartrate dehydrogenase  hypothetical protein  putative oxidoreductase  hypothetical protein  putative SAM-dependent methyltransferase  putative ATPase involved in chromosome partitioning  hypothetical protein  anaerobic C4-dicarboxylate transporter |
| InDel (Deletion) | 3191865  2862881  2154986  2862507  2864732  2033357  4577366  1381322  2967138  4566505  41565  149682  4460452  4012046  2236742  2513798 | crotonobetaine/carnitine-CoA ligase  hypothetical protein  putative cysteine dioxygenase type I  hypothetical protein  hypothetical protein  putative nitrite reductase  IS3 family element, transposase orfA  putative acyl-CoA N-acyltransferase  hypothetical protein  hypothetical protein  RelE family toxin-antitoxin system  putative transposase  hypothetical protein  hypothetical protein  amidohydrolase  putative amidase |
| Deletion | 4483105-4483135  4285993  4285745  4249761, 220090  3116597  3823463  3797863, 3719656  3459253  3214016, 2680536, 1238603, 2125737  3121070-3121095  3066035  1995008  1806922  1243734  463015 | Transporter, transcriptional regulator, alcohol /mannitol dehydrogenase  Integrase, transposase IS3/IS911 family protein  transposase InsC  OrfB, hypothetical protein  transglycosylase SLT domain protein, antirepressor protein, 20 hypothetical proteins  transcriptional regulator LuxR family, RmbA, NUDIX hydrolase, 7 hypothetical protein  OrfB, hypothetical protein  integrase family protein, putative reverse transcriptase, putative DinI-like damage-inducible protein, terminase, putative phage proteins  Transposase, hypothetical protein  putative oxidoreductase, metabolite transport protein  transcriptional regulatory protein, putative transmembrane transporter, putative tartrate dehydrogenase, putative hydroxypyruvate reductase  13 hypothetical protein, 12 putative bacteriophage proteins, lytic transglycosylase, putative reverse transcriptase, DinI family protein  tRNA-Asn, integrase, MFS superfamily transporter signal transducer, permease and ATP-binding protein of yersiniabactin-iron ABC transporter YbtQ, lipoprotein inner membrane ABC-transporter, AraC-type transcriptional regulator, yersiniabactin siderophore biosynthetic protein, pesticin/yersiniabactin TonB-dependent receptor, putative type IV secretory pathway virB1/B4/B8/B9/B10/B11 component, putative MobB/MobC mobilization protein, putative Antirestriction protein ardC, putative Retron-type reverse transcriptase  site-specific integrase gp27/gp31/gp36, putative exonuclease CP81, putative reverse transcriptase, bacteriophage sos operon Tum protein, various putative phage proteins  phage terminase, Roi protein, phage transcriptional regulator AlpA, integrase |
| Insertion | 5129192-5133570  4791092-4792539  4700723-4704895  4577699-4584461  3968213-3974320  3565402-3566728  2968052-3025250  2847405-2857366  2574980-2576581  2498954-2500276  997823-1000308  1539451-1541360  132479-133657 | Inulin fructotransferase, sugar transporter, LacI family transcriptional regulator  OrfB, transposase for insertion sequence element ISKpn1  major facilitator superfamily (MFS), Glycoside hydrolase family protein 42, DNA-binding transcriptional regulator AraC  conjugal transfer protein TraD, integrating conjugative element membrane protein%2C PFL_4697 family, ISBp1 transposase B family protein, transposase IS3/IS911 family protein  hypothetical protein, site-specific recombinase, phage integrase family, putative dTDP-glucose pyrophosphorylase  Transposase for insertion sequence element IS1328  DNA polymerase V subunit UmuC/UmuD, Formate/nitrite family of transporters, terminase small subunit, drug/metabolite transporter (DMT) superfamily permease, Phage integrase family site-specific recombinase, antimicrobial peptide transporter subunit, permease component of ABC superfamily transporter  acetylornithine deacetylase, FAD-dependent oxidoreductase, Putative bacterial extracellular solute-binding protein  Transposase  mutator family protein transposase  propanediol diffusion facilitator, bacterial regulatory helix-turn-helix, AraC family protein  group II intron reverse transcriptase/maturase  gluconolactonase |
| Complex InDels | 4803900-4805384  4120836-4125478 | plasmid stabilization protein  Site-specific recombinase XerC |
|  | 1199953-1231971 | fimbrial protein, Type VII secretion system, outer membrane protein, amino acid ABC transporter ATPase, bacterial regulatory helix-turn-helix, lysR family protein |
