# Supplementary figures and images for: The Genetic Structures of an Extensively Drug Resistant (XDR) Klebsiella pneumoniae and Its Plasmids
Source: Front Cell Infect Microbiol. 2019 Jan 4;8:446. doi: 10.3389/fcimb.2018.00446 (PMC6328971; doi:10.3389/fcimb.2018.00446)

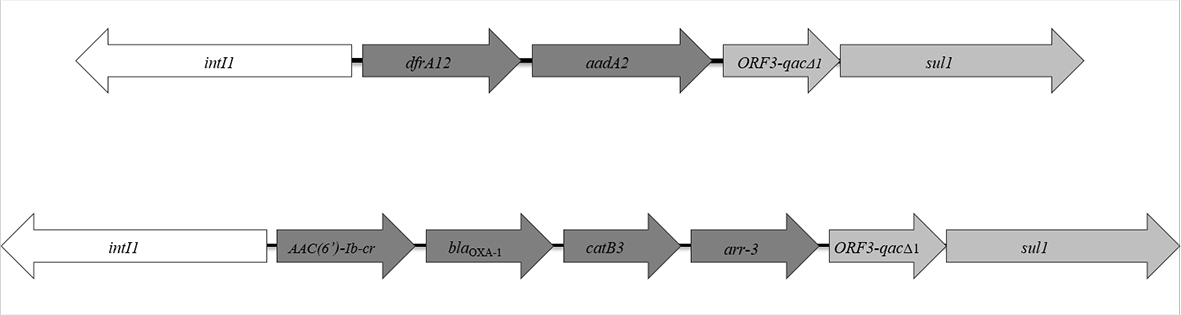

Supplement: Supplementary file 4 [file Image_1.TIF]
